# Supplementary material for: Single-cell RNA sequencing demonstrates the molecular and cellular reprogramming of metastatic lung adenocarcinoma
Source: Nat Commun. 2020 May 8;11:2285. doi: 10.1038/s41467-020-16164-1 (PMC7210975; doi:10.1038/s41467-020-16164-1)
Supplement: Supplementary file 1 — Supplementary Information [file 41467_2020_16164_MOESM1_ESM.pdf]

Supplementary Information

Single cell RNA sequencing demonstrates the molecular and cellular reprogramming of metastatic lung adenocarcinoma

Nayoung Kim et al.

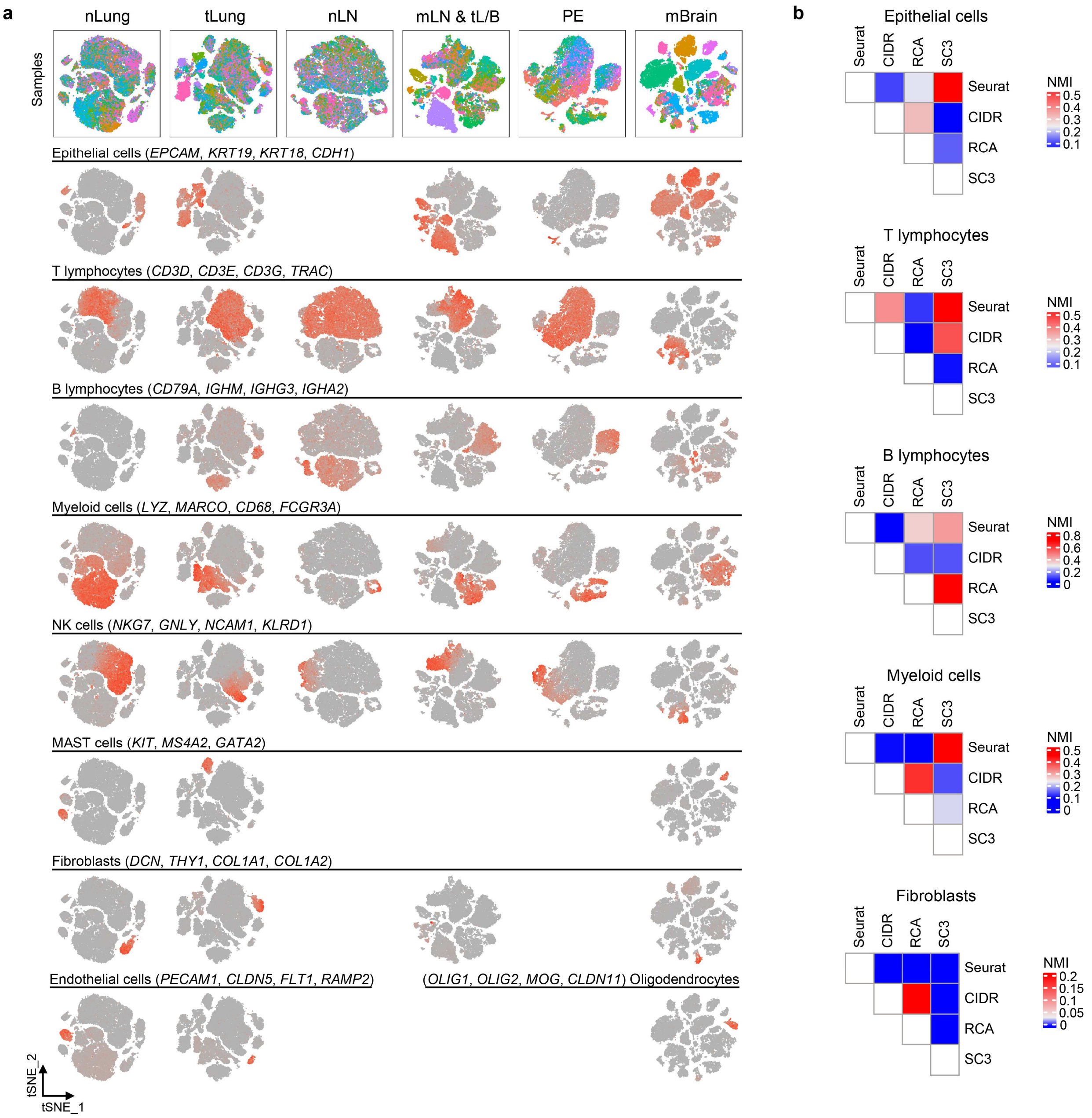

Supplementary Fig. 1. Clustering of 208,506 single cells from LUAD patients.

a, tSNE projection within tissues of each origin as in Fig. 1b, color-coded by each sample and mean expression (grey to red) of canonical marker genes for nine major lineages. **b,** Concordance for cell lineage identity based on cell clusters defined using different clustering methods in mLN & tL/B samples. The maps are colored by pairwise normalized mutual information (NMI) from four clustering methods: Seurat, CIDR, RCA, and SC3. Red and blue colors represent high and low concordance, respectively.

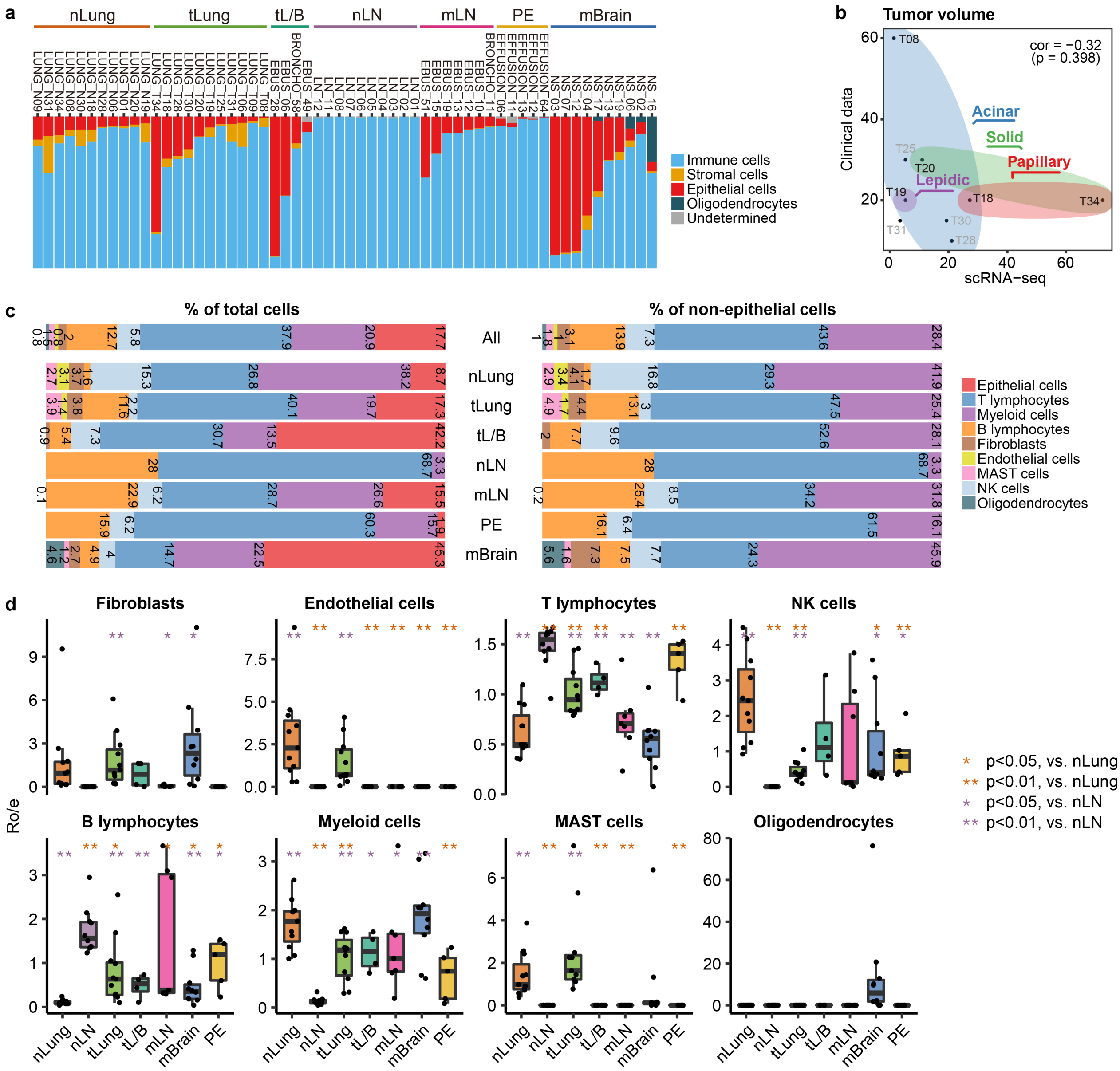

Supplementary Fig. 2. Relative proportion of nine major lineages from the tissues of each origin.

a, Overestimation of immune cells in scRNA-seq data. **b,** Comparison of cancer cell proportions in tLung estimated from scRNA-seq and clinical data. Color represents the histological types of LUAD. Cor, Pearson's correlation coefficient; p, two-sided test for the probability of a correlation. **c,** Changes in proportions of cancer and stromal components according to tissue origin. Relative proportions of major cell lineages (excluding undetermined cells) in all single (left) and non-epithelial cells (right) within tissues of each origin and all tissues. **d,** Tissue preference of the nine major lineages. $R_{O/E}$ is the relative score of observed cell numbers over expected cell numbers calculated by chi-square test. The $R_{O/E}$ values of all tissue origins are shown in different colors. Black dots represent different patients. *$p < 0.05$; **$p < 0.01$, two-sided Student's t-test. Each box represents the interquartile range (IQR, the range between the 25th and 75th percentile) with the mid-point of the data, whiskers indicate the upper and lower value within 1.5 times the

IQR. nLung, n=11 samples; tLung, n=11 samples; tL/B, n=4 samples; nLN, 10 samples; mLN, n=7 samples; PE, n=5 samples, mBrain, n=10 samples.

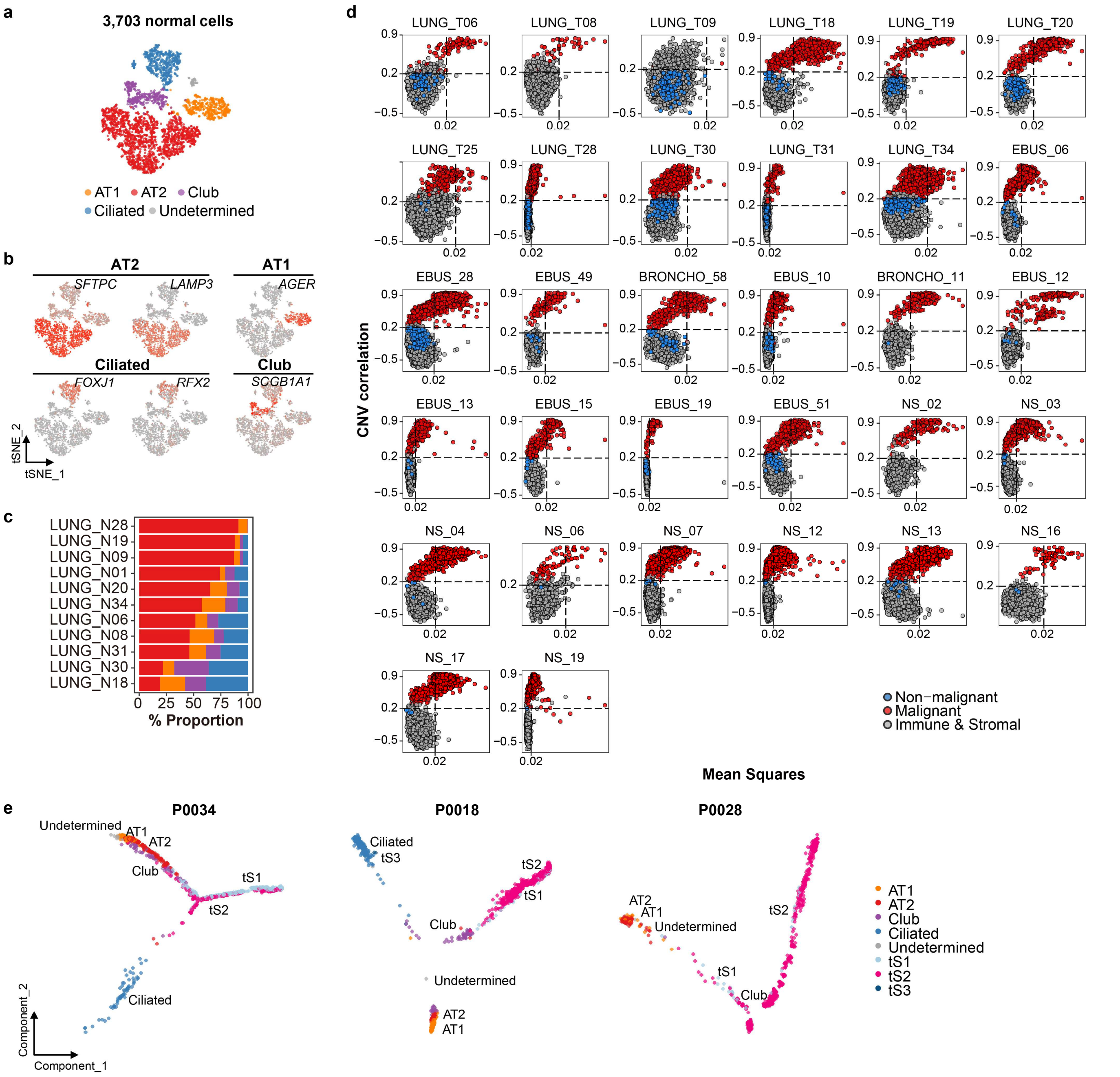

Supplementary Fig. 3. Analysis of normal epithelial cell subsets and malignant cells.

a, b, tSNE plot of normal epithelial cells in nLung, color-coded by cell subsets and expression (grey to red) of marker genes. **c,** Relative proportion of normal epithelial subsets in each sample (excluding undetermined cells). AT1, n=530 cells; AT2, n=2,020 cells; Ciliated, n=654 cells; Club, n=439 cells. **d,** Clarification of malignant cancer cells based on CNV inference. The two-dimensional (2D) plot between the mean squares and correlation of the CNV signal for each sample. Red and blue colors represent single cells defined as malignant and non-malignant, respectively. Grey represents immune and stromal cells. **e,** Unsupervised trajectory of malignant and normal epithelial cell state transition in each patient (P0034, P0018, and P0028). Colors in dots represent tumor cell states and normal epithelial cell subsets.

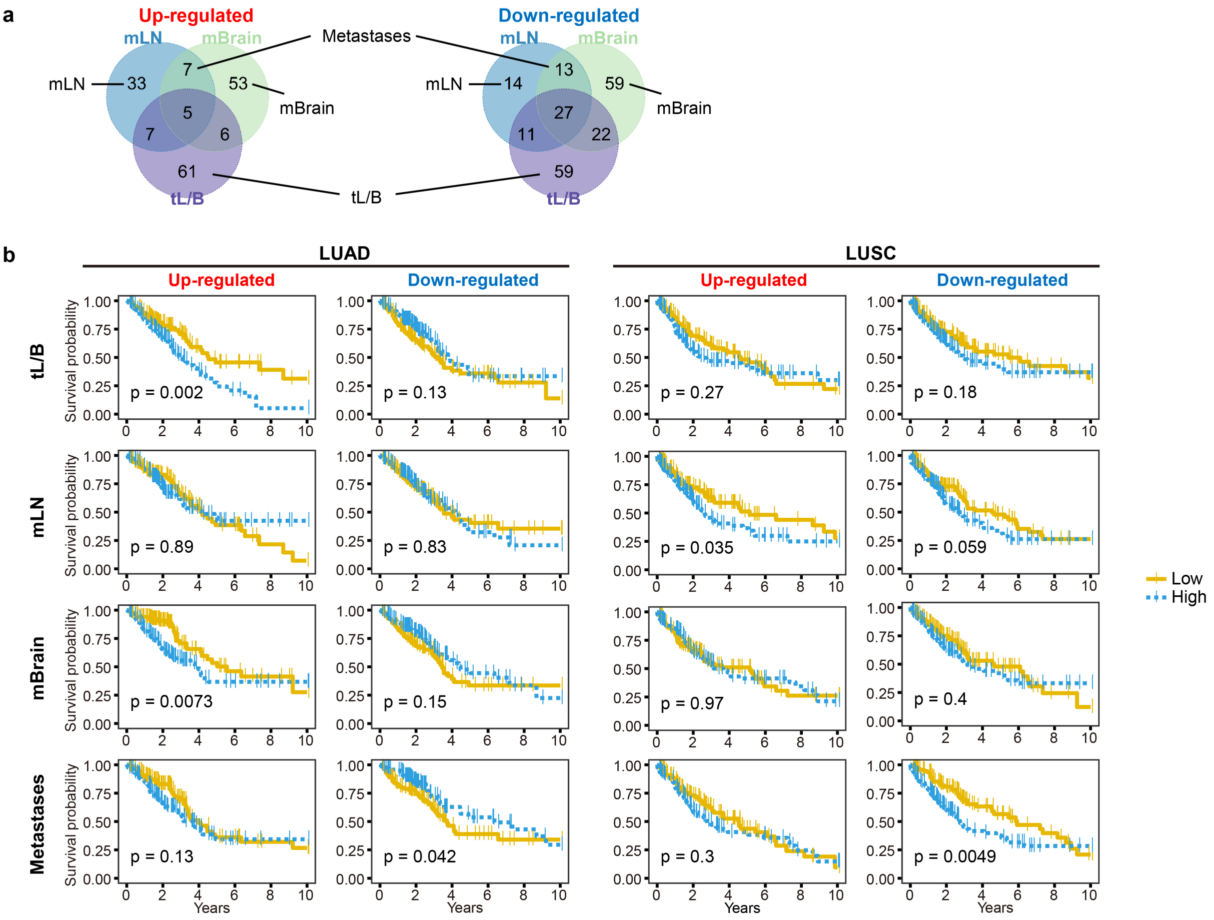

Supplementary Fig. 4. Pairwise comparison of tumor cells between tL/B, mLN, and mBrain.

a, Venn diagram of selected genes specific to tL/B, mLN, mBrain, and metastasis samples compared to tLung. **B,** Kaplan-Meier overall survival curves of TCGA LUAD (n=494 samples) and LUSC (n=490 samples) patients. + represents censored observations, and the p-value (p) was calculated through the two-sided log-rank test.

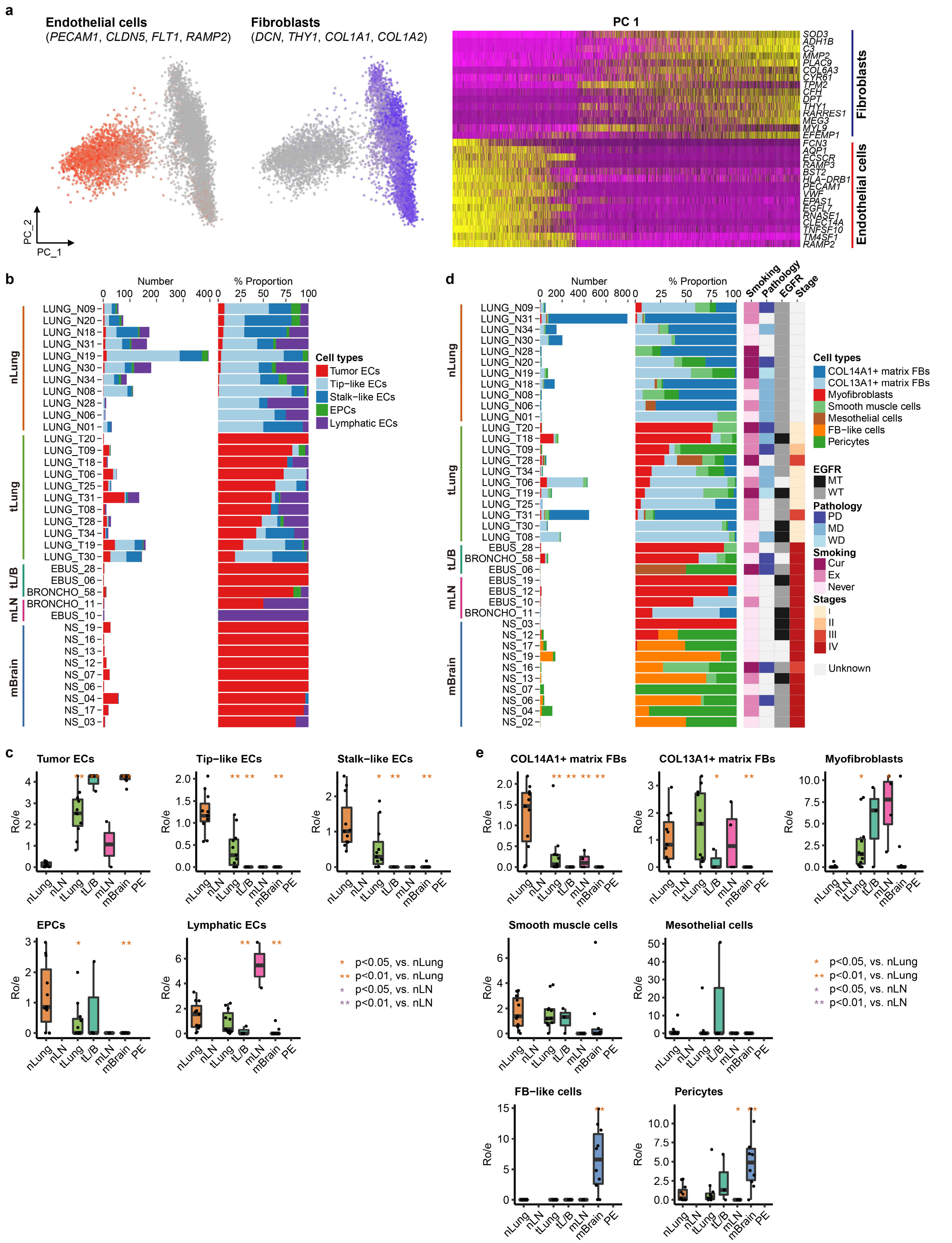

Supplementary Fig. 5. Endothelial cell and fibroblast subsets.

a, Re-classification of stromal cells into endothelial cells and fibroblasts. Unsupervised PCA on the transcriptome, indicating a distinct distribution of endothelial cells and fibroblasts (left). Individual cells are

colored red and blue to indicate the mean expression of their respective canonical markers. Expression heatmap of principal component (PC) 1 (right). Both cells and the top 30 genes are sorted by their scores for PC1. **b,** Cell number and relative proportion of endothelial cell (EC) subsets in each sample. **c,** Tissue preference of EC subsets. $R_{O/E}$ is the relative score of observed cell numbers over expected cell numbers calculated by chi-square test. The $R_{O/E}$ values of all tissue origins are shown in different colors. Black dots represent different patients. *$p < 0.05$; **$p < 0.01$, two-sided Student's t-test. nLung, n=11 samples; tLung, n=11 samples; tL/B, n=3 samples; mLN, n=2 samples; mBrain, n=9 samples. **d,** Cell number and relative proportion of fibroblast subsets (left) in each sample (excluding undetermined cells), and the association with clinical parameters (right). Number in brackets indicates the total number of fibroblasts (FB) in each sample. Cur: current smoker; Ex: ex-smoker; Never: never smoked; PD: poorly differentiated; MD: moderately differentiated; WD: well-differentiated; MT: mutant; WT: wild-type. **e,** Tissue preference of fibroblast subsets. $R_{O/E}$ is the relative score of observed cell numbers over expected cell numbers calculated by chi-square test. The $R_{O/E}$ values of all tissue origins are shown in different colors. Black dots represent different patients. *$p < 0.05$; **$p < 0.01$, two-sided Student's t-test. nLung, n=11 samples; tLung, n=11 samples; tL/B, n=3 samples; mLN, n=4 samples; mBrain, n=10 samples. In the box plot in c and e, each box represents the interquartile range (IQR, the range between the 25th and 75th percentile) with the mid-point of the data, whiskers indicate the upper and lower value within 1.5 times the IQR.

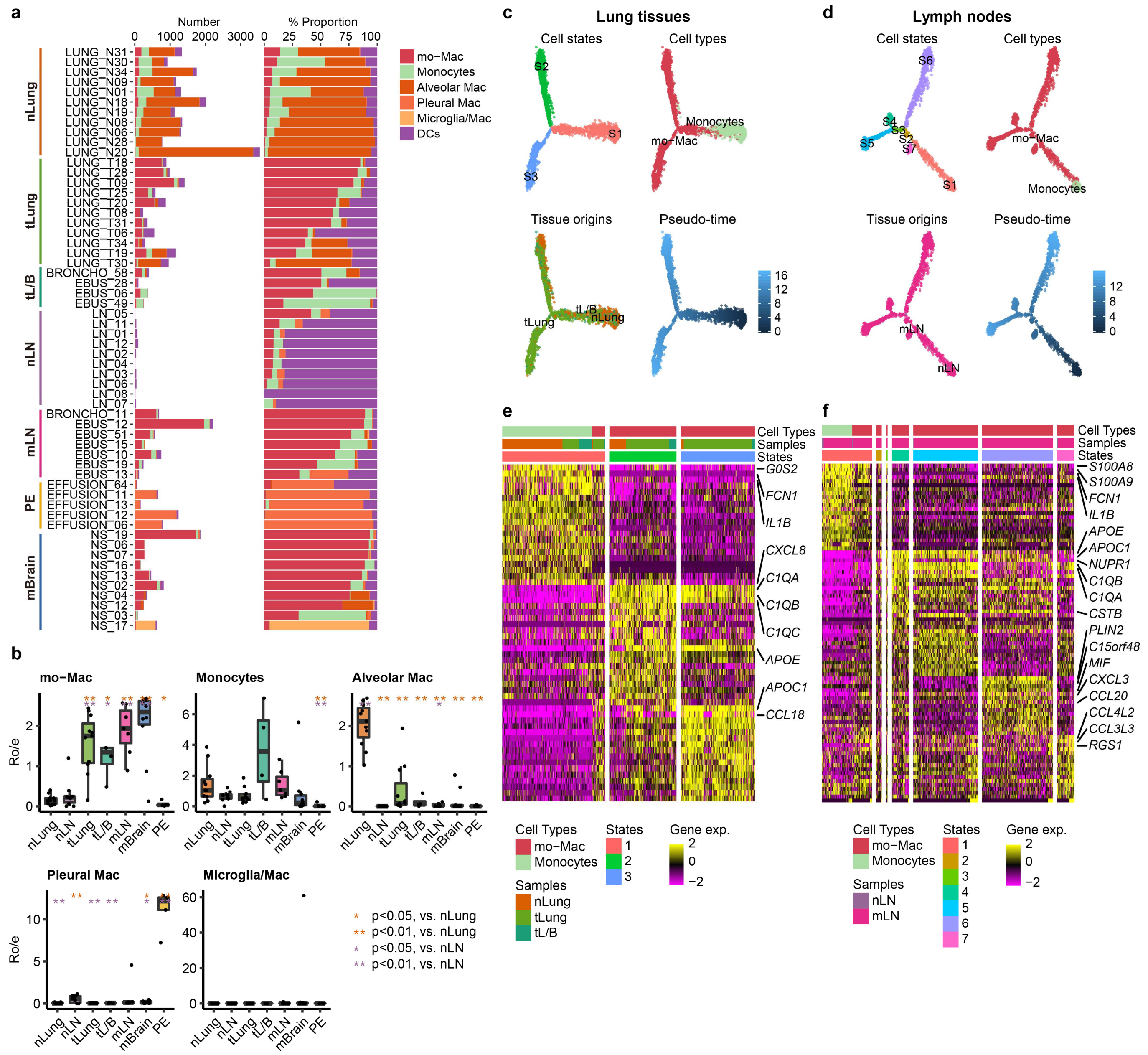

Supplementary Fig. 6. Myeloid cell subsets and analysis of monocyte and mo-Mac state transitions.

a, Cell number and relative proportion of myeloid cell subsets in each sample (excluding undetermined cells). **b,** Tissue preference of myeloid cell subsets. $R_{O/E}$ is the relative score of observed cell numbers over expected cell numbers calculated by chi-square test. The $R_{O/E}$ values of all tissue origins are shown in different colors. The black dots represent different patients. *p < 0.05; **p < 0.01, two-sided Student's t-test. Each box represents the interquartile range (IQR, the range between the 25th and 75th percentile) with the mid-point of the data, whiskers indicate the upper and lower value within 1.5 times the IQR. nLung, n=11 samples; tLung, n=11 samples; tL/B, n=4 samples; nLN, 10 samples; mLN, n=7 samples; PE, n=5 samples, mBrain, n=10 samples. **c, d,** Unsupervised trajectory of monocyte and mo-Mac state transitions in the lungs and lymph nodes. The branched trajectory was colored by cell states, cell subsets, tissue origins, and pseudo-

time, as indicated. Each cell state number was annotated with ‘Lung-Mac-S’ and ‘LN-Mac-S’, respectively. **e, f,** Relative expression map of top 20 upregulated genes specific to cell states as in Supplementary Fig. 6c, d. Expression of genes in each cell cluster is scaled by mean-centering and transformed to a scale from -2 to 2. The most expressed genes in each state is indicated on the right.

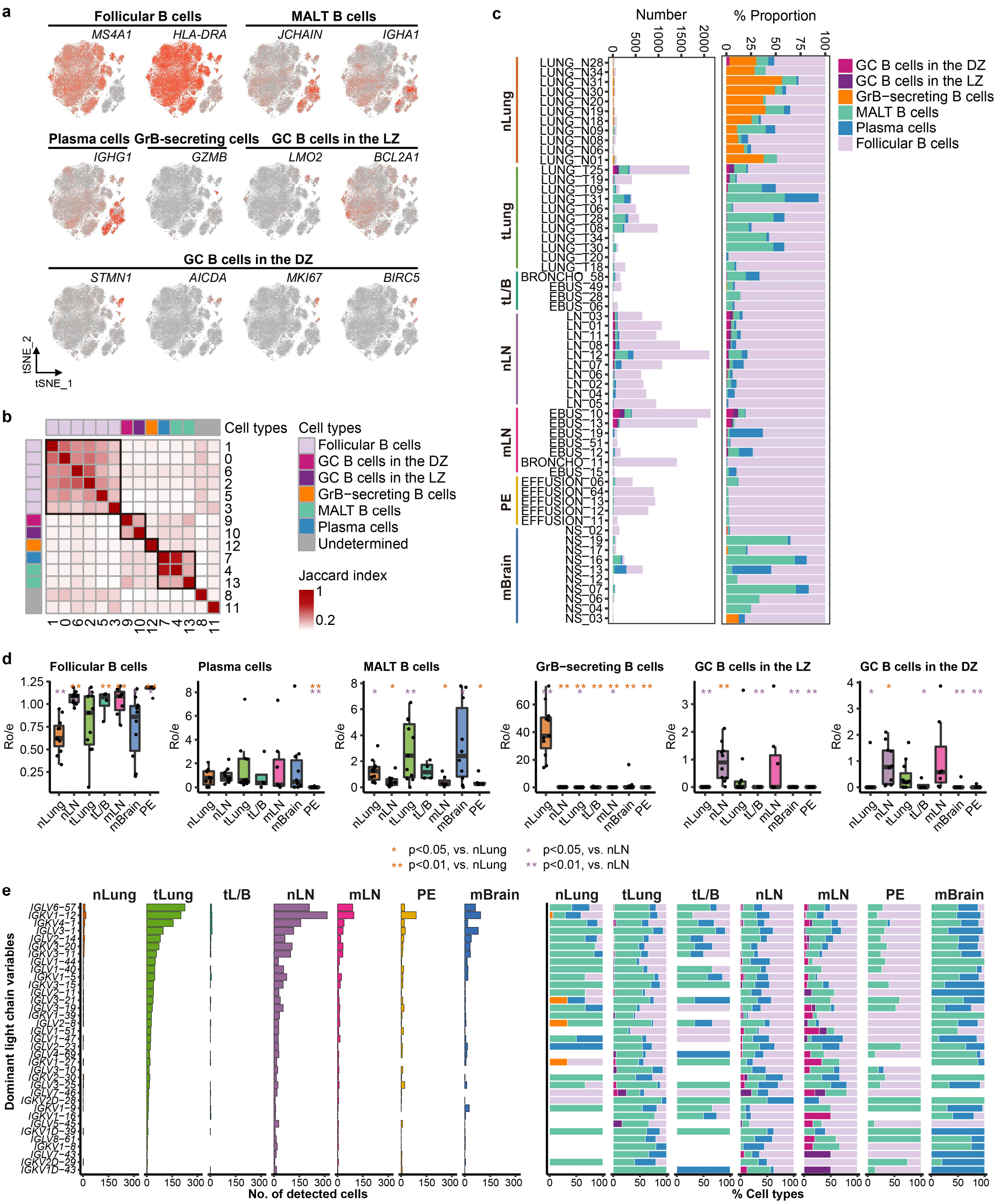

Supplementary Fig. 7. B cell subsets.

a, tSNE plot of B cells as in Fig. 5a, color-coded by their canonical marker gene expressions (grey to red). **b,** Similarity map using overlapped significant (differentially expressed genes) DEGs per B cell cluster. Color indicates Jaccard index between DEGs ($log_2FC>1$, two-sided Student's t-test p-value<0.01, adjusted p-value (Bonferroni)<0.01, and PCT>0.25). **c,** Cell number and relative proportion of B cell subsets in each sample (excluding undetermined cells). **d,** Tissue preference of B cell subsets. $R_{O/E}$ is the relative score of observed

cell numbers over expected cell numbers calculated by chi-square test. The $R_{O/E}$ values of all tissue origins are shown in different colors. The black dots represent different patients. *$p < 0.05$; **$p < 0.01$, two-sided Student's t-test. Each box represents the interquartile range (IQR, the range between the 25th and 75th percentile) with the mid-point of the data, whiskers indicate the upper and lower value within 1.5 times the IQR. nLung, n=11 samples; tLung, n=11 samples; tL/B, n=4 samples; nLN, 10 samples; mLN, n=7 samples; PE, n=5 samples, mBrain, n=10 samples. **e,** Frequency of B cells expressing immunoglobulin (Ig) light chain variable genes in our collection and B cell subset distribution in these single cells. A total of 178 immunoglobulin light chain (IgL) variable region genes were analyzed to profile the predominance and diversity of IgL in the B cell population. The dominant variable region for B cells was determined by evaluating genes with the highest expression levels. We excluded single cells with more than two regional genes showing identical expression levels. A total of 5,292 B cells with an expression of light chain variable genes were used for this analysis. Only 35 genes having expression across > 20 B cells were selected to compare the frequency and B cell subset distribution in our collection. B cell subset distribution (excluding undetermined cells) in cell groups classified according to immunoglobulin (Ig) light chain gene expression.

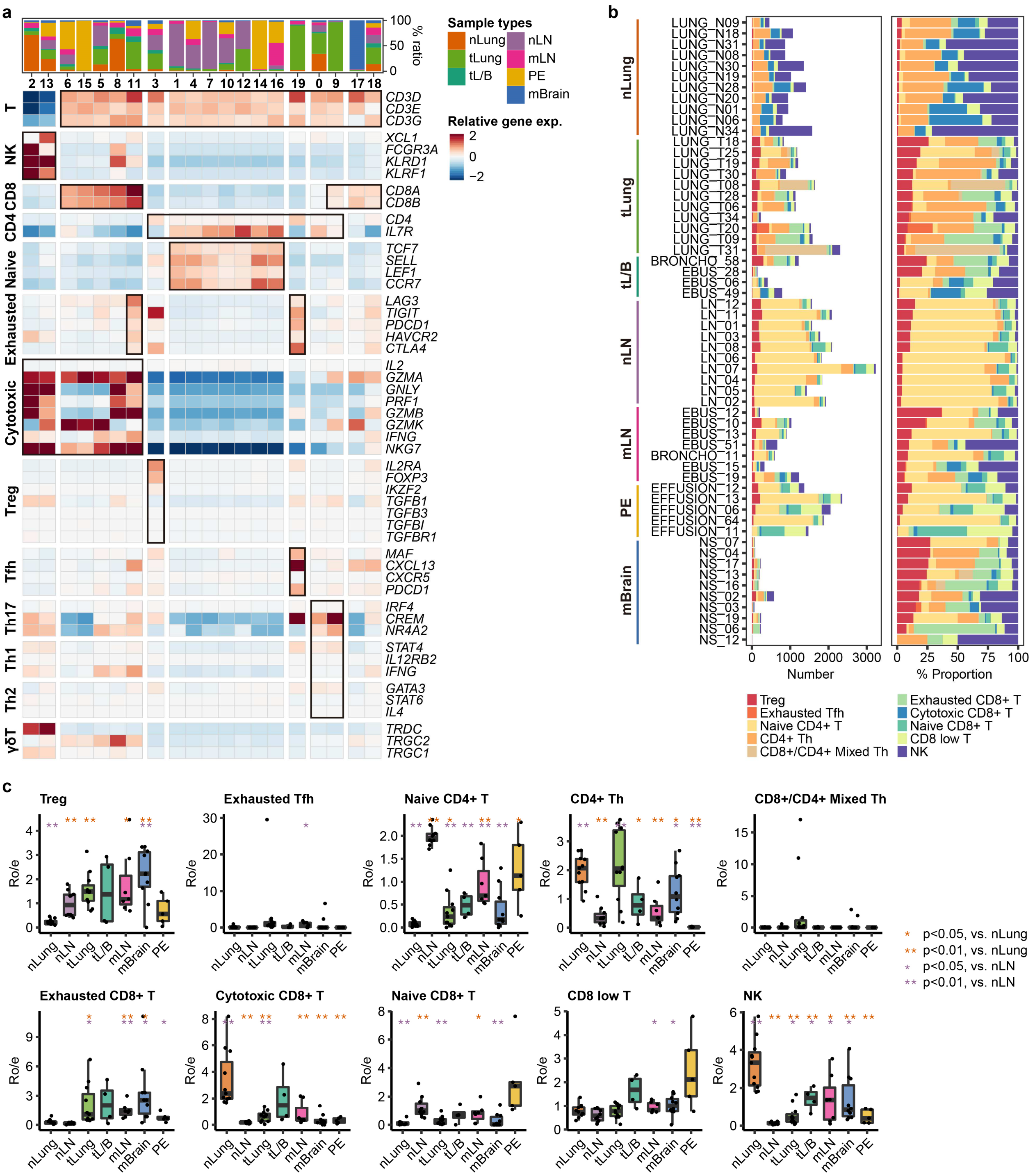

Supplementary Fig. 8. T cell subsets.

a, Tissue preference and relative mean expression map of selected marker genes associated with T cell functionality in each cell cluster. Mean expression of these genes in each cell cluster was scaled by mean-centering, and transformed into a scale from -2 to 2. **b,** Cell number and relative proportion of T cell subsets in each sample (excluding undetermined cells). **c,** Tissue preference of T cell subsets. $R_{O/E}$ is the relative score of observed cell numbers over expected cell numbers calculated by chi-square test. $R_{O/E}$ of all tissue origins is shown in different colors. The black dots represent different patients. *p < 0.05; **p < 0.01, two-sided Student's t-test. Each box represents the interquartile range (IQR, the range between the 25th and 75th

percentile) with the mid-point of the data, whiskers indicate the upper and lower value within 1.5 times the IQR. nLung, n=11 samples; tLung, n=11 samples; tL/B, n=4 samples; nLN, 10 samples; mLN, n=7 samples; PE, n=5 samples, mBrain, n=10 samples.

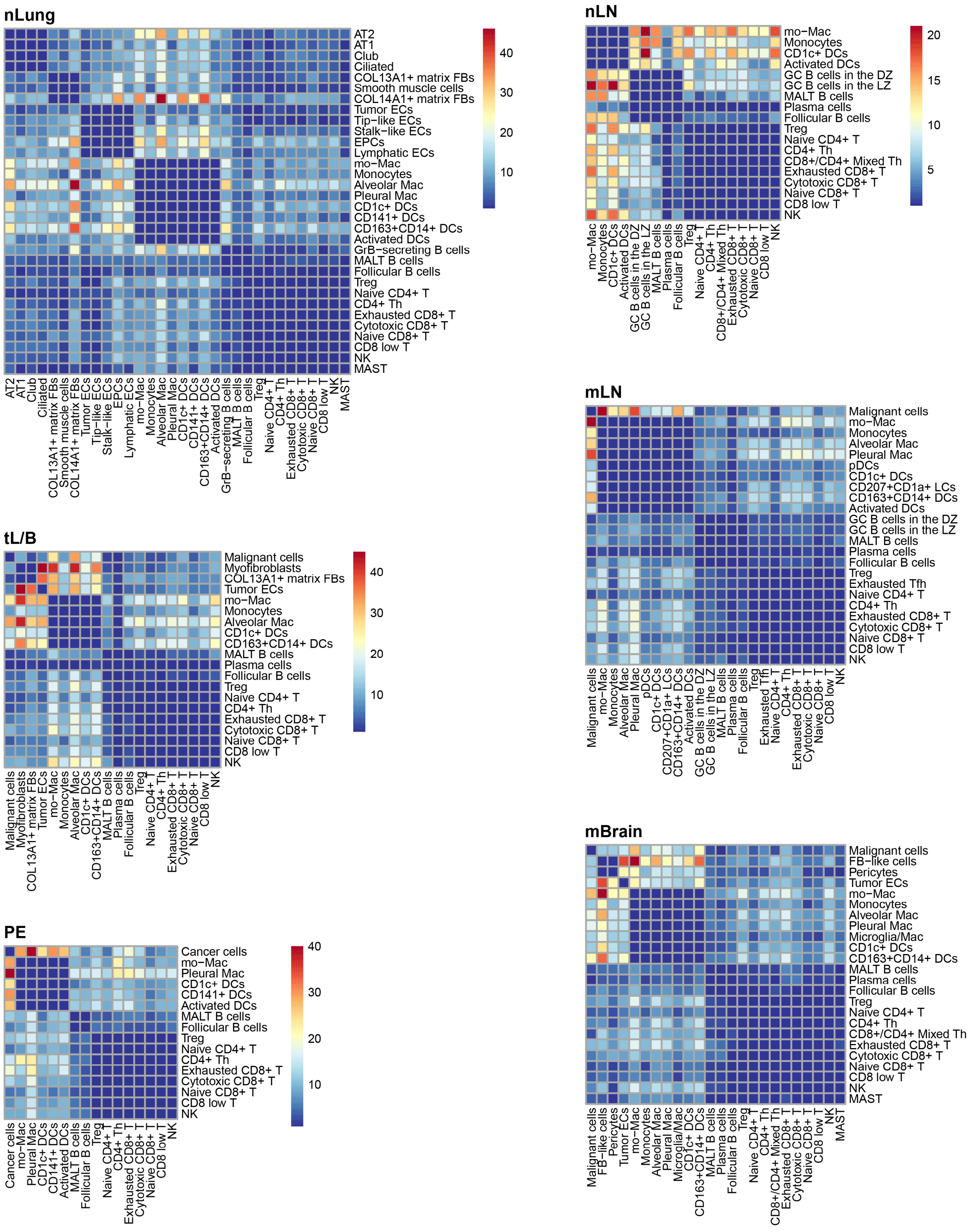

Supplementary Fig. 9. Interaction map between cell subsets within tissues of each origin.

Heat map depicting the number of significant interactions between the cell subsets in our collections except for tLung.

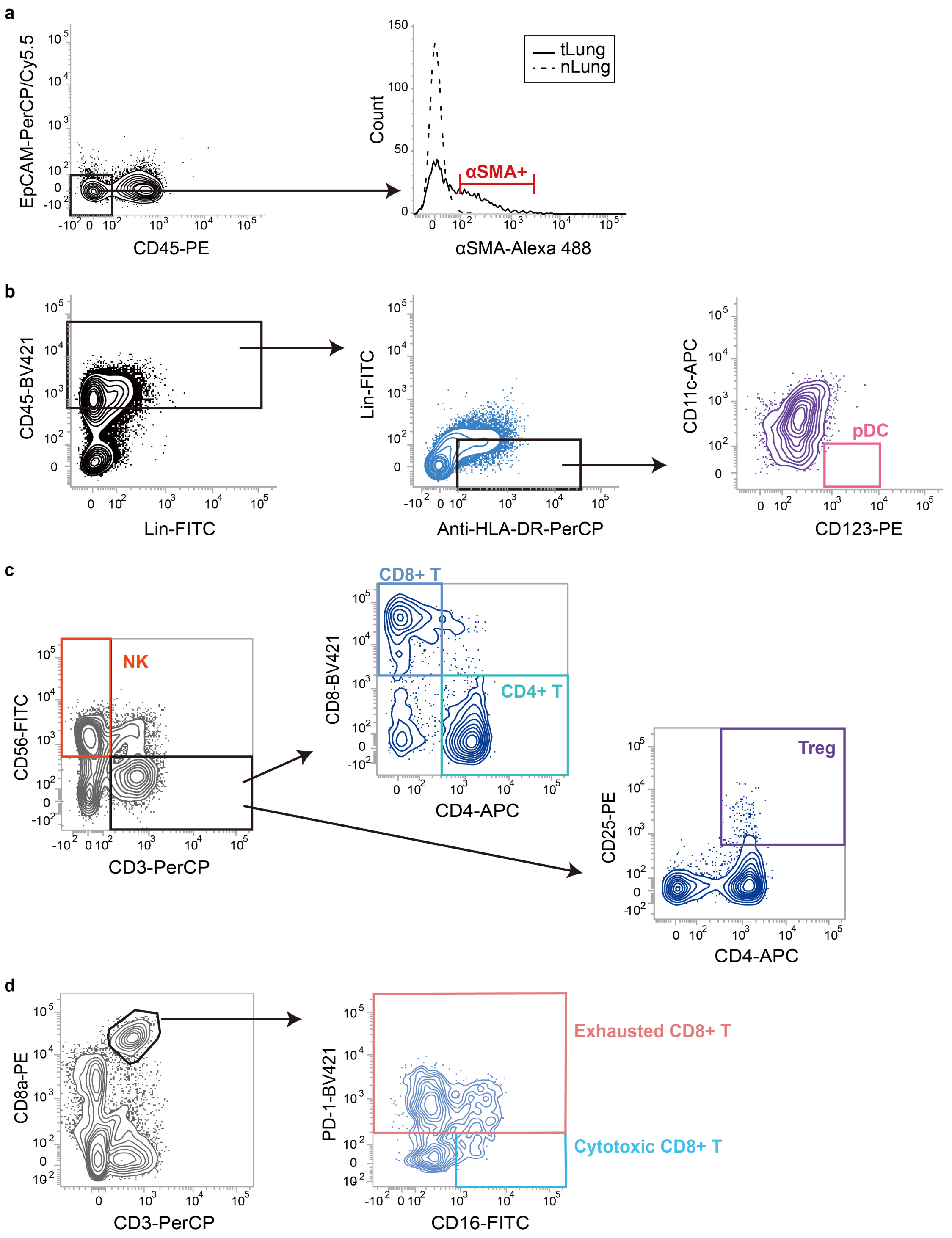

Supplementary Fig. 10. Gating strategies used for flow cytometry analysis in lung tissues.

a, Gating strategy to sort myofibroblasts (CD45+EpCAM-aSMA+) presented on Fig. 3i and j. **b,** Gating strategy to sort pDC (CD45+Lin-HLA-DR+CD11c-cd123+) cells presented on Fig. 4j and k. **c,** Gating strategy to sort NK (CD3-CD56+), CD8+ T (CD3+CD56-CD4-CD8+), CD4+ T (CD3+CD56-CD4+CD8-), and Treg (CD3+CD56-CD4+CD25+) cells presented on Fig. 5h. **d,** Gating strategy to sort cytotoxic T (CD3+CD8+CD16+PD-1-) and exhausted T (CD3+CD8+CD16-PD-1+) cells presented on Fig. 5h.
