# Supplementary material for: Single-cell RNA sequencing demonstrates the molecular and cellular reprogramming of metastatic lung adenocarcinoma
Source: Nat Commun. 2020 May 8;11:2285. doi: 10.1038/s41467-020-16164-1 (PMC7210975; doi:10.1038/s41467-020-16164-1)
Supplement: Supplementary file 3 — Description of Additional Supplementary Files [file 41467_2020_16164_MOESM3_ESM.pdf]

# Description of Additional Supplementary Files

**Supplementary Data 1. Feature summary of the lung adenocarcinoma specimens and single-cell RNA sequencing data.**

**Supplementary Data 2. List of canonical markers to assign the cell types and their references.**

**Supplementary Data 3. List of genes specific to the states (S1, S2, and S3) of malignant and normal epithelial cells including club cells and their functional categories. p-value for genes, two-sided Student's t-test; p-value for GO terms, one-sided Fisher's exact test.**

**Supplementary Data 4. List of genes specific to tL/B, mLN, mBrain, and metastasis samples. p-value, two-sided Student's t-test.**

**Supplementary Data 5. Tumor EC-specific gene lists identified at single-cell resolution and their functional categories. p-value for genes, two-sided Student's t-test; p-value for GO terms, one-sided Fisher's exact test.**

**Supplementary Data 6. List of genes specific to the states of monocytes/mo-Macs in lung tissues or lymph nodes. p-value, two-sided Student's t-test.**

**Supplementary Data 7. Full list of genes specific to the states of CD8+ T cells. p-value, two-sided Student's t-test.**

**Supplementary Data 8. Full list of ligand-receptor pairs used in the interaction analysis within tissues of each origin. One-sided p-value calculated from permutation test.**
