# Supplementary material for: Single-cell RNA sequencing demonstrates the molecular and cellular reprogramming of metastatic lung adenocarcinoma
Source: Nat Commun. 2020 May 8;11:2285. doi: 10.1038/s41467-020-16164-1 (PMC7210975; doi:10.1038/s41467-020-16164-1)
Supplement: Supplementary file 12 — Reporting Summary [file 41467_2020_16164_MOESM12_ESM.pdf]

Corresponding author(s): Myung-Ju Ahn, Hae-Ock Lee

Last updated by author(s): Mar 24, 2020

Reporting Summary

Nature Research wishes to improve the reproducibility of the work that we publish. This form provides structure for consistency and transparency in reporting. For further information on Nature Research policies, see Authors & Referees and the Editorial Policy Checklist .

Statistics

For all statistical analyses, confirm that the following items are present in the figure legend, table legend, main text, or Methods section.

n/a	Confirmed	
☐	☒	The exact sample size (*n*) for each experimental group/condition, given as a discrete number and unit of measurement
☐	☒	A statement on whether measurements were taken from distinct samples or whether the same sample was measured repeatedly
☐	☒	The statistical test(s) used AND whether they are one- or two-sided<br>*Only common tests should be described solely by name; describe more complex techniques in the Methods section.*
☒	☐	A description of all covariates tested
☐	☒	A description of any assumptions or corrections, such as tests of normality and adjustment for multiple comparisons
☐	☒	A full description of the statistical parameters including central tendency (e.g. means) or other basic estimates (e.g. regression coefficient) AND variation (e.g. standard deviation) or associated estimates of uncertainty (e.g. confidence intervals)
☐	☒	For null hypothesis testing, the test statistic (e.g. *F*, *t*, *r*) with confidence intervals, effect sizes, degrees of freedom and *P* value noted<br>*Give P values as exact values whenever suitable.*
☒	☐	For Bayesian analysis, information on the choice of priors and Markov chain Monte Carlo settings
☒	☐	For hierarchical and complex designs, identification of the appropriate level for tests and full reporting of outcomes
☐	☒	Estimates of effect sizes (e.g. Cohen's *d*, Pearson's *r*), indicating how they were calculated

Our web collection on statistics for biologists contains articles on many of the points above.

Software and code

Policy information about availability of computer code

Data collection	No specific code and software were used for data collection. The data depicted in this manuscript was generated by us.
Data analysis	Analysis performed in R version 3.4, Seurat v2.3.4, CIDR, RCA, SC3, Monocle v2, DAVID, Cytoscape v3.5.1, CellPhoneDB, CellRanger v 2.1.0, BWA-0.7.17, Picard version picard-tools-2.18.2-SNAPSHOT, GATK v4.0.5.1, and FACSuite v1.2.<br><br>Code for CNV inference is available on GitHub (https://github.com/SGI-LungCancer/SingleCell).

For manuscripts utilizing custom algorithms or software that are central to the research but not yet described in published literature, software must be made available to editors/reviewers. We strongly encourage code deposition in a community repository (e.g. GitHub). See the Nature Research guidelines for submitting code & software for further information.

Data

Policy information about availability of data

All manuscripts must include a data availability statement. This statement should provide the following information, where applicable:

- Accession codes, unique identifiers, or web links for publicly available datasets
- A list of figures that have associated raw data
- A description of any restrictions on data availability

All raw single-cell RNA sequencing and bulk WES data are available via controlled access in the European Genome-phenome Archive database (accession code EGAD00001005054), and processed data can be accessed from the NCBI Gene Expression Omnibus database (accession code GSE131907 [https://www.ncbi.nlm.nih.gov/geo/query/acc.cgi?acc=GSE131907]). Single-cell expression data can also be interactively explored online at http://ureca-singlecell.kr.
The results shown here are in part based upon data generated by the TCGA Research Network: https://www.cancer.gov/tcga.

Field-specific reporting

Please select the one below that is the best fit for your research. If you are not sure, read the appropriate sections before making your selection.

☒ Life sciences ☐ Behavioural & social sciences ☐ Ecological, evolutionary & environmental sciences

For a reference copy of the document with all sections, see nature.com/documents/nr-reporting-summary-flat.pdf

Life sciences study design

All studies must disclose on these points even when the disclosure is negative.

Sample size

Sample size for 10x Genomics scRNA-seq was determined by the availability of patient samples.
No statistical tests were performed for sample size calculation but it was sufficient for this proof-of-concept study.

The exact number of samples used per figure is informed in each figure. In the whole manuscript, they are:
11 lung tumor resection tissues (fresh)
11 normal lung resection tissues (fresh)
7 metastatic lymph node bronchoscopic biopsies (fresh)
10 normal lymph node resection tissues (fresh)
4 lung tumor bronchoscopic biopsies (fresh)
5 Pleural effusion samples (fresh)
10 metastatic brain resection tissues (fresh)

Data exclusions

All criteria for data exclusion were pre-established.

We applied three general quality measures on raw gene-cell-barcode matrix for each cell:
mitochondrial genes ≤20%,
unique molecular identifiers (UMIs) from 100 to 150,000,
gene count from 200 to 10,000

For each batch, we used the filtered cells to remove genes that are expressed at low levels by counting the number of cells (min.cells) having expression of each gene i, and excluded genes with min.cells < 0.1% cells.

Cancer cells (Figure 2 and associated Supplementary Figures) : We selected cancer cells showing perturbation in their CNV signal (>0.02 mean squares or >0.2 CNV correlation), which was classified as malignant.

Stromal cells (Figure 3 and associated Supplementary Figures) : In addition to the general QC metrics, we reclassified cells into groups of stromal cell types (2,107 endothelial cells and 3,794 fibroblasts) with concordant expression of marker genes (average log-normalized expression > 1).

T/NK cells (Figure 5 and associated Supplementary Figures) : In addition to the general QC metrics, we applied secondary cell filtration (based on the mRNA abundance of their pan-markers) to 91,227 pre-defined T/NK cells in these clusters. After the second cell filtration with concordant expression of marker genes (average of log-normalized expression > 2), 64,403 cells were confidently defined as T/NK cells.

Replication

We have replicated the specific expression of tS2 cancer cell markers and a-SMA (myofibroblasts) using immunohistochemical staining in 16 lung adenocarcinoma (LUAD) patients and one BioBank specimen, respectively. And we have replicated the different proportion of myofibroblasts, pDCs, and NK/T cell subsets (Treg, cytotoxic and exhausted CD8+ T cells) between tumor and normal lung using flow cytometry in five, four, and one LUAD patients, respectively. All replications were successful. Further analysis of larger cohorts are recommended as future work.

Randomization

The patients with lung adenocarcinoma were recruited randomly in this study.

Blinding

Not applicable since there was no specific grouping.

Reporting for specific materials, systems and methods

We require information from authors about some types of materials, experimental systems and methods used in many studies. Here, indicate whether each material, system or method listed is relevant to your study. If you are not sure if a list item applies to your research, read the appropriate section before selecting a response.

Materials & experimental systems

n/a	Involved in the study
[ ]	[x] Antibodies
[x]	[ ] Eukaryotic cell lines
[x]	[ ] Palaeontology
[x]	[ ] Animals and other organisms
[ ]	[x] Human research participants
[x]	[ ] Clinical data

Methods

n/a	Involved in the study
[x]	[ ] ChIP-seq
[ ]	[x] Flow cytometry
[x]	[ ] MRI-based neuroimaging

Antibodies

Antibodies used

Antibody (IHC) Clone Source Catalog#
anti-IGFBP3 MM0342-6U23 Novus Biologicals Cat# NBP2-12364
anti-CK19 Polyclonal Novus Biologicals Cat# NB100-687
anti-AG2 Polyclonal Novus Biologicals Cat# NBP2-27393
anti-S100a2 EPR5392 Abcam Cat# ab109494
anti-aSMA 1A4 DAKO Agilent Cat# M0851

Antibody (Flow cytometry) Clone Dilution Source Catalog#
CD56-FITC HCD56 1:20 BioLegend Cat# 318303
CD3-PerCP UCHT1 1:20 BioLegend Cat# 300428
CD4-APC/Cy7 OKT4 1:20 BioLegend Cat# 317417
CD8-BV421 SK1 1:20 BioLegend Cat# 344747
CD25-PE BC96 1:20 BioLegend Cat# 302605
CD8a-PE HIT8a 1:20 BioLegend Cat# 300908
CD16-FITC B73.1 1:20 BioLegend Cat# 360715
PD-1-BV421 EH12.2H7 1:20 BioLegend Cat# 329920
EpCAM-PerCP/Cy5.5 EBA-1 1:5 BD Biosciences Cat# 347199
CD45-PE HI30 (RUO) 1:5 BD Biosciences Cat# 555483
aSMA-Alexa 488 E184 1:50 Abcam Cat# ab197240
CD45-BV421 2D1 1:20 BioLegend Cat# 368521
Anti-Human 4-Color Dendritic Value Bundle BD Biosciences Cat# 340565
- Lineage Cocktail 1-FITC Polyclonal 1:5 BD Biosciences Cat# 340546
- Anti-HLA-DR-PerCP L243 1:10 BD Biosciences Cat# 347364
- CD11c-APC S-HCL-3 1:20 BD Biosciences Cat# 340544
- CD123-PE 9F5 1:20 BD Biosciences Cat# 340545

Validation

All the antibodies used in this study were commercial antibodies, with validation procedures described on the following sites of the manufacturers:

Antibody (IHC) Clone Source Catalog#
anti-IGFBP3 MM0342-6U23 Novus Biologicals Cat# NBP2-12364
- https://www.novusbio.com/products/igfbp-3-antibody-mm0342-6u23_nbp2-12364
- Validated for immunohistochemistry (IHC) in paraffin-embedded human tissues by the manufacturer.
anti-CK19 Polyclonal Novus Biologicals Cat# NB100-687
- https://www.novusbio.com/products/cytokeratin-19-antibody_nb100-687
- Validated for immunohistochemistry (IHC) in paraffin-embedded human tissues by the manufacturer.
anti-AG2 Polyclonal Novus Biologicals Cat# NBP2-27393
- https://www.novusbio.com/products/ag-2-agr2-antibody_nbp2-27393
- Validated for immunohistochemistry (IHC) in paraffin-embedded human tissues by the manufacturer.
anti-S100a2 EPR5392 Abcam Cat# ab109494
- https://www.abcam.com/s100-alpha-2s100a2-antibody-epr5392-ab109494.html
- Validated for immunohistochemistry (IHC) in paraffin-embedded human tissues by the manufacturer.
anti-aSMA 1A4 DAKO Agilent Cat# M0851
- https://www.agilent.com/en/product/immunohistochemistry/antibodies-controls/primary-antibodies/actin-(smooth-muscle)-(concentrate)-76542#support
- Optimized for immunohistochemistry (IHC) with validated protocols by the manufacturer. This antibody has been cited by more than 199 publications.

Antibody (Flow cytometry) Clone Dilution Source Catalog#
CD56-FITC HCD56 1:20 BioLegend Cat# 318303
- https://www.biolegend.com/it-it/products/fitc-anti-human-cd56-ncam-antibody-3795

- Flow cytometry was tested on human peripheral blood lymphocytes by the manufacturer.
CD3-PerCP UCHT1 1:20 BioLegend Cat# 300428
- https://www.biolegend.com/it-it/products/percp-anti-human-cd3-antibody-4213
- Flow cytometry was tested on human peripheral blood lymphocytes by the manufacturer.
CD4-APC/Cy7 OKT4 1:20 BioLegend Cat# 317417
- https://www.biolegend.com/it-it/products/apc-cyanine7-anti-human-cd4-antibody-3658
- Flow cytometry was tested on human peripheral blood lymphocytes by the manufacturer.
CD8-BV421 SK1 1:20 BioLegend Cat# 344747
- https://www.biolegend.com/it-it/products/brilliant-violet-421-anti-human-cd8-antibody-13512
- Flow cytometry was tested on human peripheral blood lymphocytes by the manufacturer.
CD25-PE BC96 1:20 BioLegend Cat# 302605
- https://www.biolegend.com/it-it/products/pe-anti-human-cd25-antibody-616
- Flow cytometry was tested on human peripheral blood lymphocytes by the manufacturer.
CD8a-PE HIT8a 1:20 BioLegend Cat# 300908
- https://www.biolegend.com/it-it/products/pe-anti-human-cd8a-antibody-762
- Flow cytometry was tested on human peripheral blood lymphocytes by the manufacturer.
CD16-FITC B73.1 1:20 BioLegend Cat# 360715
- https://www.biolegend.com/it-it/products/fitc-anti-human-cd16-antibody-9302
- Flow cytometry was tested on human peripheral blood lymphocytes by the manufacturer.
PD-1-BV421 EH12.2H7 1:20 BioLegend Cat# 329920
- https://www.biolegend.com/it-it/products/brilliant-violet-421-anti-human-cd279-pd-1-antibody-7191
- Flow cytometry was tested on human peripheral blood lymphocytes by the manufacturer.
EpCAM-PerCP/Cy5.5 EBA-1 1:5 BD Biosciences Cat# 347199
- https://www.bdbiosciences.com/eu/applications/research/stem-cell-research/cancer-research/mouse/percp-cytrade55-mouse-anti-human-epcam-eba-1/p/347199
- Flow cytometry was tested on human sample by the manufacturer.
CD45-PE HI30 (RUO) 1:5 BD Biosciences Cat# 555483
- https://www.bdbiosciences.com/eu/applications/research/stem-cell-research/cancer-research/human/pe-mouse-anti-human-cd45-hi30/p/555483
- Flow cytometry was tested on human peripheral blood lymphocytes by the manufacturer.
aSMA-Alexa 488 E184 1:50 Abcam Cat# ab197240
- https://www.abcam.com/alpha-smooth-muscle-actin-antibody-e184-alexa-fluor-488-ab197240.html
- Flow cytometry was tested on human cell line (HeLa) by the manufacturer.
CD45-BV421 2D1 1:20 BioLegend Cat# 368521
- https://www.biolegend.com/it-it/products/brilliant-violet-421-anti-human-cd45-antibody-14686
- Flow cytometry was tested on human peripheral blood lymphocytes by the manufacturer.
Anti-Human 4-Color Dendritic Value Bundle BD Biosciences Cat# 340565
- https://www.bdbiosciences.com/us/reagents/research/clinical-research---ruo-gmp/multicolor-cocktails/4---color-cocktails/anti-human-4-color-dendritic-value-bundle/p/340565
- Flow cytometry was tested on human peripheral blood lymphocytes by the manufacturer.

Human research participants

Policy information about studies involving human research participants

Population characteristics: Forty-four patients with pathological LUAD diagnosis were enrolled in this study. None of the patients had received prior treatment. Detailed information can be found in the Human specimens section of Methods and Supplementary Data 1.

Recruitment: All donors are recruited and managed by Samsung Medical Center, avoiding the selection of poorly clinically characterized (unclear diagnosis for cancer subtypes) volunteers.

Ethics oversight: Lung adenocarcinoma patients; The medical ethics committee of the Institutional Review Board (IRB) of Samsung Medical Center

Note that full information on the approval of the study protocol must also be provided in the manuscript.

Flow Cytometry

Plots

Confirm that:

- [x] The axis labels state the marker and fluorochrome used (e.g. CD4-FITC).
- [x] The axis scales are clearly visible. Include numbers along axes only for bottom left plot of group (a 'group' is an analysis of identical markers).
- [x] All plots are contour plots with outliers or pseudocolor plots.
- [x] A numerical value for number of cells or percentage (with statistics) is provided.

Methodology

Sample preparation	Dissociated cells were multi-stained with three to five antibodies at 4 °C for 1 h, and then washed once with phosphate buffered saline. The cells were analyzed after filtering through a round-bottom tube with a 40-µm strainer-cap.
Instrument	FACSVerse (BD Biosciences)
Software	FACSuite v1.2 (BD Biosciences)
Cell population abundance	N/A. No sorting performed. Flowcytometry was performed for analytical purposes.
Gating strategy	Supplementary Fig. 10 to graphically account for all FACS sequential gating/sorting strategies

☒ Tick this box to confirm that a figure exemplifying the gating strategy is provided in the Supplementary Information.
